# Clinical therapeutic effects of anterior decompression on spinal osteoporotic fracture and inflammatory cytokines

**DOI:** 10.12669/pjms.305.5369

**Published:** 2014

**Authors:** Qi Liao, Shi-Qing Liu, Jiang-Hua Ming, Qing Chen, Qi Zhao, Yue Yang

**Affiliations:** 1 Qi Liao, Department of Orthopedics, Renmin Hospital, Wuhan University, 238 Jiefang Road, Wuhan 430060, Hubei Province, P. R. China.; 2 Shi-Qing Liu, Department of Orthopedics, Renmin Hospital, Wuhan University, 238 Jiefang Road, Wuhan 430060, Hubei Province, P. R. China.; 3 Jiang-Hua Ming, Department of Orthopedics, Renmin Hospital, Wuhan University, 238 Jiefang Road, Wuhan 430060, Hubei Province, P. R. China.; 4 Qing Chen, Department of Orthopedics, Renmin Hospital, Wuhan University, 238 Jiefang Road, Wuhan 430060, Hubei Province, P. R. China.; 5 Qi Zhao, Department of Orthopedics, Renmin Hospital, Wuhan University, 238 Jiefang Road, Wuhan 430060, Hubei Province, P. R. China.; 6 Yue Yang, Department of Orthopedics, Renmin Hospital, Wuhan University, 238 Jiefang Road, Wuhan 430060, Hubei Province, P. R. China.

**Keywords:** Anterior decompression, Spinal osteoporotic fracture, Inflammatory cytokine, complication

## Abstract

***Objective:*** To evaluate the clinical therapeutic effects of anterior decompression on spinal osteoporotic fracture and inflammatory cytokines.

***Methods:*** A total of 140 patients with spinal osteoporotic fracture were selected and randomly divided into a treatment group and a control group (n=70). The control group was treated by central corpectomy, and the control group was treated by anterior decompression.

***Results:*** The rate of excellent and good outcomes in the treatment group was 94.3%, and that of the control group was 78.6%, which differed significantly (P<0.05). Cobb angle and cord occupancy in the spinal canal of both groups significantly decreased (P<0.05), while height ratio of the injured vertebral body significantly increased (P<0.05). Meanwhile, there were statistically significant inter-group differences (P<0.05). During the three-month follow-up period, the treatment group was significantly less prone to complications such as superficial infection, spinal instability and screw breakage compared with the control group (P<0.05). The postoperative serum MMP-3 and IL-6 levels of both groups significantly decreased compared with those before surgeries (P<0.05), with statistically significant inter-group differences (P<0.05).

***Conclusion: ***Compared with central corpectomy, anterior decompression exerted better effects on spinal osteoporotic fracture by improving the prognosis and stabilizing the spine safely, which may be associated with the effectively reduced serum MMP-3 and IL-6 levels.

## INTRODUCTION

Senior diseases are spouting recently with population aging. Osteoporotic fracture, as one of the common diseases endangering middle-aged and elderly people, affects their quality of life. Particularly, osteoporosis-induced spinal fracture is most common, which is closely related with the high load and large movement range of spine.^1^^,^^2^ Besides the signs of fracture, there are also symptoms such as intermittent claudication due to nerve root, horse-tail and vascular compressions.^3^^,^^4^ Spinal osteoporotic fracture surgeries are performed for spinal decompression that is conventionally treated by laminectomy or hemi-semi laminectomy.^5^ Although the surgeries are advantageous in sufficient decompression and clear vision, removing most of the dorsal columns of the spinal cord affects the prognosis and recovery of patients by jeopardizing the stability and biomechanics of spine.^6^

Currently, anterior decompression has been widely applied to treat cervical myelopathy involving lower than two segments, but its application in treating spinal fracture remains controversial.^7^ Many types of inflammatory cytokines can be detected in the synovial fluid of spinal osteoporotic fracture patients who suffer from immune regulation disorders and functional changes of many types of immune cells.^8^ As a pro-inflammatory cytokine, interleukin (IL)-6 has been extensively studied in the subject matter of spinal osteoporotic fracture. Human matrix metalloproteinases (MMPs), which are zinc-dependent endonucleases, function predominantly in the degradation and rebuilding of extracellular matrix.^9^ As a member of the MMPs family, MMP-3 is secreted by synovial cells, chondrocytes and endothelial cells, thus playing an important role in the destruction of bone and articular cartilage by substantially degrading extracellular matrix.^10^ In this study, the therapeutic effects of anterior decompression on spinal osteoporotic fracture and inflammatory cytokines were evaluated.

## METHODS

***Subjects: ***A total of 140 patients with spinal osteoporotic fracture treated in our hospital from September 2010 to November 2013 were included in this study which was approved by the institutional ethics committee, and written consent was obtained from all patients. 

***Inclusion criteria:*** In accordance with the diagnosis standards of thoracolumbar vertebral fracture (confirmed by X-ray film, CT-scanning and MRI scanning results); onset within two weeks; with clinical symptoms such as lumbago, backache, lower extremity pain, numbness and paralysis of the lower limbs; 40-80 years old; suitable for surgical treatment. 

***Exclusion criteria:*** Congenital absence of cervical pedicles; spinal fracture complicated with damages of vital organs. The patients comprised 76 males and 64 females with the average age of (59.34 ± 3.21) years old (42~78 years old). 

***Causes of injury***: 40 cases of fall injury, 78 cases of traffic accident injury, 12 cases of crush injury, and another 20 cases. This included 4 cases of explosion-induced fractures, 10 cases of fighting-induced fractures, and 6 cases of exercise-induced fractures. 

***Fracture sites:*** 80 cases of T_1_ segment, 25 cases of T_12_ segment, 5 cases of L_11_ segment, and 35 cases of L_2_ segment. 

***Frankel classification for spinal cord injury:*** 10 cases of Grade B, 25 cases of Grade C, 35 cases of Grade D, and 60 cases of Grade E. The patients were randomly divided into a treatment group and a control group (n=70). The gender, age, injury causes, fracture sites and Frankel classification of the two groups were similar (P>0.05).

***Treatment methods: ***Control group: Central corpectomy was performed, after which autologous bone was implanted through the decompression window and was fixed by a titanium locking plate with appropriate length.

***Treatment group:*** Anterior decompression was performed. Under inhalation anesthesia, the angle between the trunk of patient and the horizontal plane was maintained at 60° in his/her left lateral decubitus position. After sterilization of surgical drape, the site approximately 5 cm away from the dorsomedian line was longitudinally incised with the injured spine as the center. After the fascia, trapezius and posterior serratus were cut open layer by layer, the 12th rib was found and marked as the spinal column succumbing to fracture. After the involved parapophysis was removed, 1-2 ribs near the spine were further removed and intercostal neurovascular bundles were ligated and severed. Then the damaged vertebral body, intercalated disc involved and intraspinal sclerites were explored, and those intruding in the spinal canal were gradually cleared to completely relieve the oppression.

All the patients were subjected to anti-inflammatory, dehydration and prophylactic antibiotics treatments, and were recommended to take part in functional exercise rehabilitation after conventional drainage and suture.

***Observation indices: ***Observation of therapeutic effects: Overall therapeutic effects were evaluated after surgeries. Excellent: Disappearance of all clinical symptoms with normal activities of daily living; good: mild low back pain with unrestricted activities of daily living; fair: moderate low back pain with restricted activities of daily living; poor: severe low back pain with seriously restricted activities of daily living.

***Evaluation on spinal functions:*** The Cobb angle, height ratio of the injured vertebral body and cord occupancy in the spinal canal of all patients before and after surgeries were determined and compared. Cobb angle refers to the angle of intersection between the vertical line of the upper edge of cephalad end vertebra and that of the lower edge of caudal end vertebra.

***Determination of inflammatory cytokines:*** Bloods (2-4 ml) were collected from all patients before and after surgeries, maintained still for 30 min and centrifuged at 2000 r/min for 10 min. Then the supernatant was collected and subpackaged into EP tubes that were stored at -80°C prior to use. Human MMP-3 (kit from Shanghai Senxiong Technology Industry Co., Ltd.) and human IL-6 (kit from Jingmei Biotech Co., Ltd.) levels were measured by the double antibody sandwich ELISA method according to the instructions.

***Observation of complications:*** After surgeries, all patients were followed-up for three months. The main complications included superficial infection, spinal instability and screw breakage, etc.

***Statistical analysis: ***All data were analyzed by SPSS 18.0. The numerical data were expressed as (x ± s). Inter-group and intra-group comparisons were performed by t test or analysis of variance and SNK-q test respectively. The categorical data were compared by Chi-square analysis. P<0.05 was considered statistically significant.

## RESULTS

***Overall therapeutic effects: ***The rate of excellent and good outcomes in the treatment group was 94.3%, and that of the control group was 78.6%, which differed significantly (P<0.05) (Table-I).

**Fig.1:**
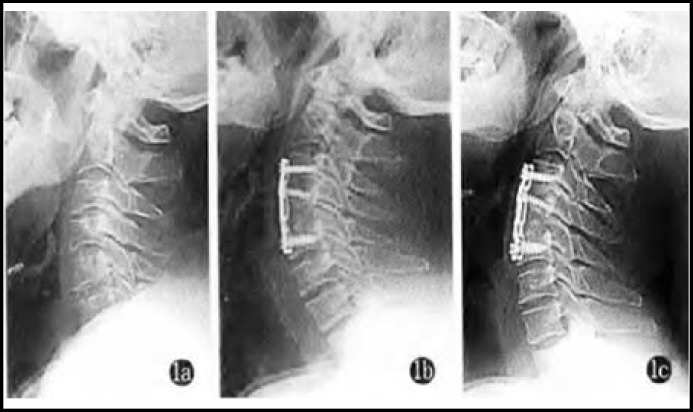
Mr. Wang, male, 62 years old, T_1_ segment. a: Preoperative X-ray film disclosed height of the injured vertebral body and Cobb angle; b: height of the injured vertebral body and Cobb angle immediately after central corpectomy; c: height of the injured vertebral body and Cobb angle three months after surgery (satisfactory maintenance and bone fusion).

**Fig.2:**
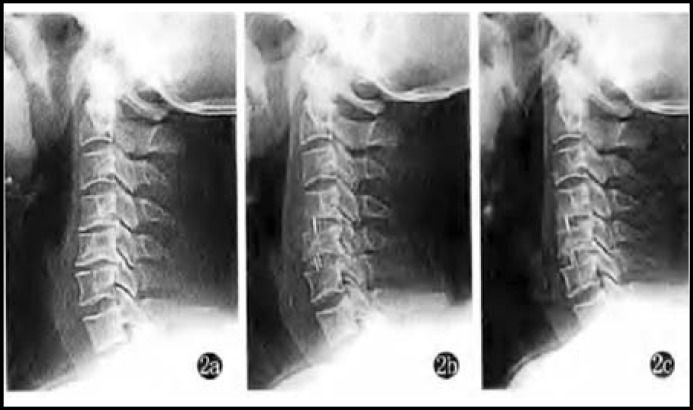
Mr. Liu, male, 62 years old, T_1_ segment. a: Preoperative X-ray film disclosed height of the injured vertebral body and Cobb angle; b: height of the injured vertebral body and Cobb angle immediately after anterior decompression; c: height of the injured vertebral body and Cobb angle three months after surgery (satisfactory maintenance and bone fusion).

***Indices of spinal functions:*** Cobb angle and cord occupancy in the spinal canal of both groups significantly reduced (P<0.05), while height ratio of the injured vertebral body significantly rose (P<0.05). In the meantime, there were statistically significant inter-group differences (P<0.05) (Table-II, Fig. 1 and Fig. 2).

***Changes of inflammatory cytokine levels: ***The postoperative serum MMP-3 and IL-6 levels of both groups significantly reduced compared with those before surgeries (P<0.05), with statistically significant inter-group differences (P<0.05) (Table-III).

***Complications: ***During the three-month follow-up period, the treatment group was significantly less prone to complications such as superficial infection, spinal instability and screw breakage compared with the control group (P<0.05) (Table-IV).

## DISCUSSION

Since the anatomic structure of spine is complicated and the elderly are vulnerable to osteoporosis, spinal fracture easily occurs upon severe trauma. Besides vertebral fractures, spinal osteoporotic fracture is often concomitant with vertebral attachment fractures, ligament rupture and damages to adjacent intervertebral discs, which destabilize spine as well as physically and psychologically affect the patients complicated with spinal cord injury.^11^ An epidemiological investigation showed that the incidence rate of traumatic spinal fracture increased annually in China with rapid development of modern transportation as well as industrial and agricultural construction. Generally, simple spinal fractures do not give rise to evident dysfunctions, whereas those complicated with spinal osteoporosis may lead to severe disability.^12^

**Table-I:** Overall therapeutic effects (n).

***Group***	***Case No. ***	***Excellent***	***Good***	***Fair***	***Poor***	***Rate of excellent and good outcomes***
Treatment group	70	53	13	3	1	94. 3%
Control group	70	40	15	10	5	78. 6%
χ^2^						5. 092
P						<0. 05

**Table-II:** Indices of spinal functions (x ± s).

***Index***	***Time point***	***Treatment group (n=70)***	***Control group (n=70)***	***t***	***P***
Cobb angle (°)	Before	26. 12 ± 3. 23	26. 45±6. 35	0. 342	>0. 05
	After	6. 73 ± 1. 78^	10. 80 ± 1. 88^	4. 240	<0. 05
Height ratio of the injured vertebral body (%)	Before	35. 87±4. 09	35. 89±5. 11	0. 098	>0. 05
	After	94. 56 ± 4. 03^	66. 98 ± 9. 34^	17. 832	<0. 05
Cord occupancy in the spinal canal (%)	Before	64. 56±5. 09	64. 89±6. 11	0. 124	>0. 05
	After	9. 67 ± 2. 45^	18. 98 ± 1. 78^	10. 453	<0. 05

Compared with the results before surgeries, t=19. 839, 33. 887, 21. 288, 14. 789, 15. 098, 21. 009, P<0. 05.

**Table-III:** Inflammatory cytokine levels (mmol/L, x ± s).

***Index***	***Time point***	***Treatment group (n=70)***	***Control group (n=70)***	***t***	***P***
MMP-3	Before	33. 12 ± 2. 09	34. 34 ± 3. 01	0. 453	>0. 05
	After	23. 09 ± 4. 32^	28. 12 ± 2. 54^	6. 453	<0. 05
IL-6	Before	7. 13±0. 89	7. 15±0. 45	0. 089	>0. 05
	After	1. 98 ± 0. 67^	4. 09 ± 0. 55^	5. 892	<0. 05

Compared with the results before surgeries, t=12. 123, 21. 498, 5. 998, 15. 877, P<0. 05.

**Table-IV:** Complications (n).

***Group***	***Case No. ***	***Superficial infection***	***Spinal instability***	***Screw breakage***	***Total***
Treatment group	70	1	0	1	2 (2. 9%)
Control group	70	4	3	4	11 (15. 7%)
χ^2^					3. 098
P					<0. 05

Spinal osteoporotic fracture is mainly treated by surgeries to maintain the 3D shape of spine and to provide an optimum environment for the recovery of nerves, which require proper internal fixation and decompression.^13^ Decompression is traditionally realized by laminectomy or hemi-semi laminectomy that results in lumbar spondylolisthesis and spinal instability though. Hence, the lowly traumatic and highly specific decompression is currently used.^14^

It is well-known that spinal osteoporotic fracture usually involves the anterior and center spinal columns. Although posterior decompression is advantageous in facile operation, short surgical time and less blood loss, it cannot exert effects specifically. Moreover, central decompression may lead to loss of vertebral body height, thus jeopardizing the prognosis by inducing spinal stenosis or fracture, loosening and failure of internal fixation. In contrast, anterior decompression is able to remove the obstacle oppressing the frontal spinal canal under direct vision, and to stabilize the spinal column by directly constructing the fractured vertebral bodies of the anterior and center spinal columns in cooperation with internal fixation apparatus and bone grafts.^15^ In this study, the rate of excellent and good outcomes in the treatment group was 94.3%, and that of the control group was 78.6%, which differed significantly (P<0.05). Cobb angle and cord occupancy in the spinal canal of both groups significantly decreased (P<0.05), while height of the injured vertebral body significantly increased (P<0.05). Meanwhile, there were statistically significant inter-group differences (P<0.05). The results suggested that both surgeries well promoted functional recovery. However, anterior decompression functioned better, manifested as the minimized influence on the spinal stability.

MMPs, which play essential roles in the degradation and remodeling of extracellular matrix, can be stimulated by pro-inflammatory cytokines such as IL-6 to be produced in fibroblasts, macrophages, synovial cells and chondrocytes.^16^ Particularly, MMP-3 may participate in the onset of inflammation and osteoporosis, and predominantly regulate bone destruction. The down-regulation of MMP-3 alleviates osteoporosis and destruction of cartilage and bone by decreasing the release of inflammatory cytokines and by regulating abnormal matrix degradation and angiogenesis. As a pro-inflammatory cytokine, IL-6 is highly expressed in bone fracture patients, the level of which is weakly correlated with spinal function score.^17^ In this study, the postoperative serum MMP-3 and IL-6 levels of both groups significantly decreased compared with those before surgeries (P<0.05), with statistically significant inter-group differences (P<0.05).

In the midst of surgeries for spinal fracture, patients are bound to undergo major trauma, considerable blood loss, together with complications endangering large blood vessels of the chest and abdomen and organs. Regardless, anterior decompression allows better interbody fusion and thus can prevent chronic spinal instability induced by intervertebral scar. Furthermore, the surgery can be performed easily without causing considerable bleeding.^18^ In this study, during the follow-up period, the treatment group was significantly less prone to complications such as superficial infection, spinal instability and screw breakage compared with the control group (P<0.05). The complications can be treated based on individual cases. For instance, spinal canal should be cautiously examined before placing bone cement to implant screws. The size of screws should be optimized and the superior articular process should also be carefully identified.

In summary, anterior decompression excels laminectomy in the treatment of spinal osteoporotic fracture, which improves the prognosis and stabilizes the spinal column safely. The outcomes may be associated with the effectively reduced MMP-3 and IL-6 levels.

## Authors’ Contributions:

**QL and SQL:** Study design and manuscript preparation;

**JHM, QC, QZ and YY: **Data collection and analysis, as well as manuscript preparation.
